# Spontaneous Unilateral Extracranial Internal Carotid Artery Dissection and Pseudoaneurysm Presenting as Complete Caudal Cranial Nerve Palsy

**DOI:** 10.1002/ccr3.70980

**Published:** 2025-09-24

**Authors:** Justus M. Müller‐Goebel, Nathalie Nierobisch, Hans H. Jung, Alexander Huber, Jörg E. Bohlender

**Affiliations:** ^1^ Department of Otorhinolaryngology, Head and Neck Surgery University Hospital Zurich Zurich Switzerland; ^2^ University of Zurich Zurich Switzerland; ^3^ Department of Neuroradiology, Clinical Neuroscience Center University Hospital Zurich, University of Zurich Zurich Switzerland; ^4^ Department of Neurology University and University Hospital Zurich Zurich Switzerland; ^5^ Division of Phoniatrics and Speech Pathology University Hospital Zurich Zurich Switzerland

**Keywords:** cervical artery, Collett‐Sicard, cranial nerves paresis, dissection, internal carotid artery, jugular foramen

## Abstract

In patients exhibiting multiple cranial nerve palsy, pathologies such as carotid artery dissection close to the jugular foramen should be considered as a potential cause of the rare Collett‐Sicard syndrome. Interdisciplinary collaboration helps to facilitate rapid diagnosis and treatment establishment. Conservative management with antithrombotic medication results in a satisfying outcome.

## 1 Introduction

Dissection of the cervical arteries (CA), including the extracranial internal carotid (ICA) and vertebral artery (VA), is a common cause of thromboembolic ischemic insult, especially in young adults. In the case of intracranial extension of the dissection, subarachnoid bleeding can be a possible sequel. The incidence of ICA dissections is 2.5–3/100.000 [1], with the potential to affect lower cranial nerves (CN) in 3 to 12% of cases, contingent on the nerves affected [2]. Besides dissections due to (minor) trauma, spontaneous ICA dissections may occur. Connective tissue disorders are accountable in the minority of cases [1, 2]. Due to its vicinity to certain skull base foramen, affection of CN may be a relevant guiding symptom in ICA dissections, besides ipsilateral Horner syndrome, severe headache, or stroke symptoms [2, 3]. In clinical work‐up, cerebrovascular ultrasound, computer tomography (CT), magnetic resonance imaging (MRI), and eventually digital subtraction angiography (DSA) aid in establishing the correct diagnosis; they define the location of origin and extension of the dissection [1, 2, 4]. Sometimes, an angiographic recanalization is indicated. Secondary prophylactic antithrombotic or anticoagulation medication is a treatment option of choice. Yet conclusive randomized control trials are still missing, defining which of the latter medications is superior in preventing recurrence. Nevertheless, recurrence is rare, and the risk decreases over time [1].

## 2 Case History/Examination

**FIGURE 1:**
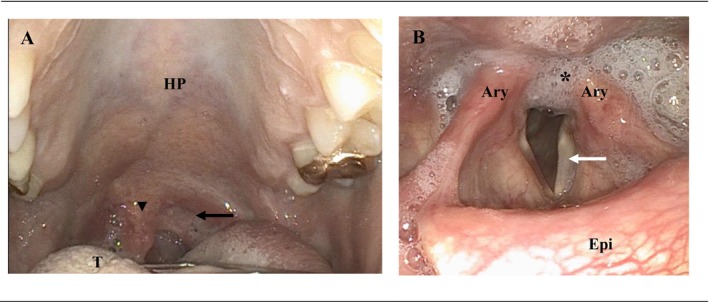
Clinical manifestation of cranial nerve paresis. (A) Oral inspection with uvula deviation to the unaffected side (arrowhead) and low tension of the anterior palatine collum (black arrow) representing vagal and glossopharyngeal paresis. T, tongue; HP, hard palate. (B) Paramedian position of the left vocal fold due to vagal nerve paresis during respiration (white arrow). Increased saliva pooling in the left piriform sinus with penetration (asterisk) and subsequent clinically elevated risk of silent aspiration. Ary, arytenoid cartilage; Epi, epiglottis.

**FIGURE 2:**
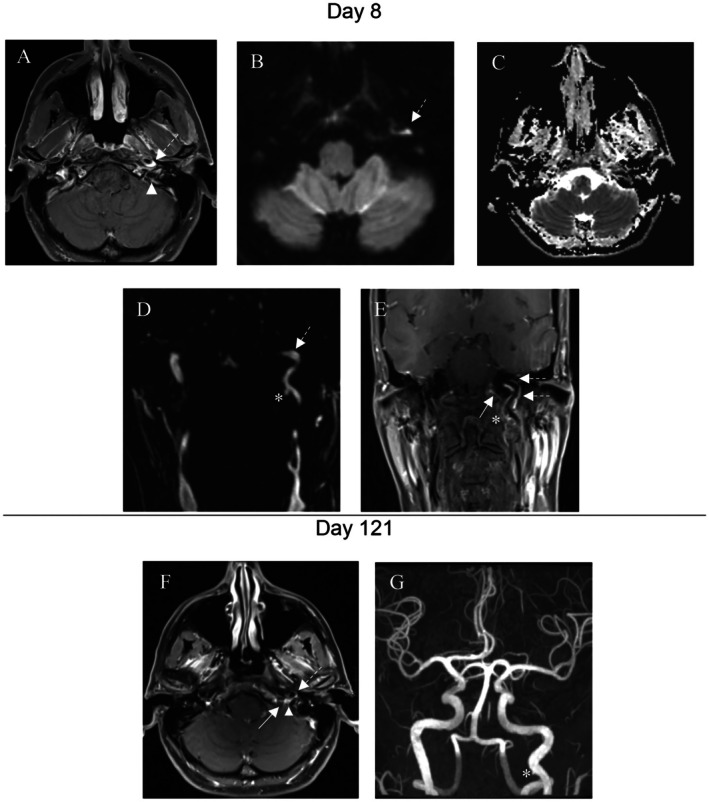
Radiological assessment at presentation and follow‐up. (A–E) Scull base MRI 8 days after symptom onset. (A) Axial imaging with T1 weighted TSE sequence and contrast media depicting jugular foramen (arrowhead) and dissection (dashed arrow). (B, C) DWI sequence showing diffusion restriction of dissection (dashed arrow), with corresponding ADC sequence. (D, E) Angiography in coronary plane with contrast media and visualization of dissection (dashed arrow) and pseudoaneurysm (asterisk) in close proximity to hypoglossal canal (white arrow). (F, G) Follow up after 121 days showing almost complete involution of both findings in axial scull base MRI. (F) T1‐weighted TSE with contrast media and (G) TOF magnet angiography. ADC, apparent diffusion coefficient; DWI, diffusion weighted imaging; MRI, magnet resonance imaging; TOF, time‐of‐flight; TSE, turbo‐spin‐echo.

A 48‐year‐old Caucasian woman presented to the emergency department of a tertiary referral center with progressing dysarthria, swallowing impairment, and insufficient left arm elevation for 3 days after a prior intense left‐sided headache. No trauma was reported. Medical history revealed a transitory ischemic attack (TIA) 7 years ago, a persisting oval foramen, and a migraine with aura. Prophylactic acetylsalicylic acid was taken daily (100 mg). Consultation at a local hospital the day before with inconspicuous neurological status and additional brain MRI did not demonstrate cerebral ischemia. Clinical examination in our emergency department revealed impairment of the glossopharyngeal (CN IX), vagal (CN X), accessory (CN XI), and hypoglossal (CN XII) CN, presenting with previously described symptoms. Due to symptom progression, an MRI of the brain was repeated with no inflammation of the caudal CN or a brainstem compressing mass being visualized. Hematologic and serologic laboratory results in combination with lumbar puncture were inconclusive. Consecutive assessment of the ear‐nose and throat (ENT) and phoniatric specialists confirmed CN impairments by detailed visualization of left vocal fold paresis with dysarthria due to vagal nerve affection (CN X), deviation of the uvula (CN IX), weakness of shoulder elevation and head rotation (CN XI), and tongue deviation (CN XII), the latter contributing to impaired oral intake (Figure 1). Additional MRI of the neck including the skull base was performed, demonstrating a dissection of the extracranial ICA with elongated intramural hematoma extending in the vicinity of the jugular foramen and hypoglossal canal as well as a medio‐caudal pseudoaneurysm (Figure 2). Neurovascular ultrasound excluded stenosis or reduced flow of the ICA; yet, it was unable to detect the dissection.

## 3 Differential Diagnosis

Alternative causes of the multiple CN pathology such as cranial polyneuritis were excluded by laboratory testing and lumbar puncture. Additional brain MRI strengthened the absence of an infectious origin and excluded an intracranial mass. Further considerable investigations in respect to connective tissue disorders such as Marfan or Ehlers‐Danlos syndrome were not undertaken, since medical history and clinical findings did not point towards such a genetic predisposition in a 48‐year‐old woman. A recurrence of a TIA was primarily not perceived to be of significance due to elapsed time and progressing symptoms.

## 4 Conclusion and Results (Outcome and Follow‐Up)

A thorough medical history, in combination with a detailed clinical examination and additional radiological imaging, is warranted to diagnose CN affection due to an ICA dissection and to initiate antithrombotic treatment to prevent stroke complications on time.

The symptoms were stable during admission; therefore, an outpatient logopedic and physiotherapy appointment was scheduled. Radiological follow‐up with MRI after 3 months showed almost complete regression of the hematoma and the pseudoaneurysm, consistent with improved clinical symptoms. A limitation of left arm elevation was the only minor impairment persistent at 4 months of clinical follow‐up, resulting in a discrete winged scapula. Physiotherapy was continued, and acetylsalicylic acid was kept for secondary prevention.

## 5 Discussion

Extracranial ICA dissections are commonly located caudally of the temporal bone due to its mobility regarding its surroundings, contrary to the otherwise anchored course in bone [1, 5]. Its proximity to penetrating CN at the jugular foramen and hypoglossal canal can lead to a local mass effect due to a resulting intramural hematoma and consecutive compression of the adjacent CN or restriction of the nerves' nutrition by compression of their *vasa vasorum* [3]. Therefore, paralysis of surrounding CN is reported in up to 12%, with the hypoglossal nerve (CN XII) being most often involved [1, 3, 6]. Notably, multiple causes can lead to a mass effect, including tumors extending to the skull base, with paraganglioma [7] being reported to be likely [6, 8]. In the presented case, radiological imaging showed a long‐distance hematoma due to spontaneous dissection of the distal part of the ICA from the atlanto‐occipital joint to the carotid canal (Figure 2) with an additional pseudoaneurysm in the more proximal course. A retrospective evaluation of the case shows consistent presentation of the Collet‐Sicard syndrome [2] consisting of complete caudal CN palsy. Its precise epidemiology in the literature remains uncertain due to the rare occurrence. The preceding intense unilateral headache reported by the patient is a common first local sign of ICA dissections [9], which could be falsely mistaken for a migraine episode given the medical history in this case. Local symptoms are typically precede the ICA dissection [9]. The described symptom progression, including complete caudal CN impairments due to expansive dissection, was most likely caused by the extension of the hematoma within the vessel wall [2]. There were no signs of Horner's syndrome, so the clinical symptoms did not fulfill the criteria of Villaret's syndrome. Cerebrovascular ultrasound did not demonstrate flow obstruction as a risk factor for thromboembolic events, nor was it able to visualize the causative aortic dissection. Furthermore, the patient was taking acetylsalicylic acid due to the history of a TIA, possibly preventing potential thrombotic formation and consecutive vessel occlusion or embolism. Alternative causes of the multiple CN pathology such as cranial polyneuritis were excluded by laboratory testing, lumbar puncture, and additional brain MRI. Further investigations in respect to connective tissue disorders such as Marfan or Ehler‐Danlos syndrome were not undertaken since medical history or clinical findings did not point towards such a genetic predisposition [1]. The further course showed a clinical and radiological improvement, as typically seen in ICA dissections within weeks to months. After 4 months of follow‐up, solely left shoulder elevation was slightly impaired without limitation in daily routine. Based on the data in the literature, the recurrence rate of spontaneous CA dissection is reported to be as low as 2% within the first months. The likelihood decreases to 1% per year over time [1].

## Author Contributions

**Justus M. Müller‐Goebel:** conceptualization, data curation, investigation, methodology, visualization, writing – original draft, writing – review and editing. **Nathalie Nierobisch:** data curation, visualization, writing – review and editing. **Hans H. Jung:** conceptualization, supervision, writing – review and editing. **Alexander Huber:** conceptualization, supervision, writing – original draft, writing – review and editing. **Jörg E. Bohlender:** conceptualization, resources, supervision, writing – original draft, writing – review and editing.

## Ethics Statement

This case report is exempt from Institutional Review Board (IRB) review, as it does not meet the criteria for human subjects' research as defined by the relevant guidelines and policies. Written informed consent was obtained from the patient to publish this report in accordance with the journal's patient consent policy.

## Consent

Written informed consent was obtained from the patient to publish this report in accordance with the journal's patient consent policy.

## Conflicts of Interest

The authors declare no conflicts of interest.

## Data Availability

The authors have nothing to report.
